# Chemotaxonomic and molecular phylogenetic studies of selected *Hypoxylon* species from the Neotropics

**DOI:** 10.1080/21501203.2024.2378071

**Published:** 2024-08-22

**Authors:** Marjorie Cedeño-Sanchez, Christopher Lambert, Luis C. Mejia, Sherif S. Ebada, Marc Stadler

**Affiliations:** a Department of Microbial Drugs, Helmholtz Centre for Infection Research (HZI) and German Centre for Infection Research, Braunschweig, Germany; b Institute of Microbiology, Technische Universität Braunschweig, Braunschweig, Germany; c Molecular Cell Biology Group, Helmholtz Centre for Infection Research (HZI), Braunschweig, Germany; d Department of Cell Biology, Helmholtz Centre for Infection Research (HZI), Braunschweig, Germany; e Centro de Biodiversidad y Descubrimiento de Drogas, Instituto de Investigaciones Científicas y Servicios de Alta Tecnología (INDICASAT–AIP), Panamá, Republic of Panama; f Smithsonian Tropical Research Institute, Apartado 2072, Balboa, Republic of Panamá; g Department of Pharmacognosy, Faculty of Pharmacy, Ain Shams University, Cairo, Egypt

**Keywords:** Epitype, phylogeny, stromatal pigments, Xylariales

## Abstract

Members of the genus *Hypoxylon* (Ascomycota) are pleomorphic fungi mostly forming conspicuous teleomorphs, consisting of perithecia embedded into stromal tissue, and their morphology has traditionally served for species delineation. However, analysis in tandem with other phenotypic characters, such as chemical and genetic traits, proved to be a more stable predictor of interspecies and intergeneric relationships. During 2014 and 2015, a set of species identified as *Hypoxylon* were described from the Neotropics, exclusively relying on morphological traits. The secondary metabolite profiles of their stromata were analysed by HPLC/DAD-ESI-MS, corroborating their classification within Xylariales. Additionally, molecular data for ex-type strains of *H. dussii* and *H. sofaiense* were incorporated into an inferred molecular phylogeny of the Hypoxylaceae and allies. Furthermore, a freshly collected specimen from North Carolina was selected as epitype of *Sphaeria perforata* Schweinitz (syn. *Hypoxylon perforatum*), as its morphological/chemotaxonomic characters matched those of the holotype. Our findings demonstrate that the secondary metabolism of *Hypoxylon* closely correlates with both morphological features and molecular data, serving as a complement for species identification.

## 1. Introduction

**Figure 1.**
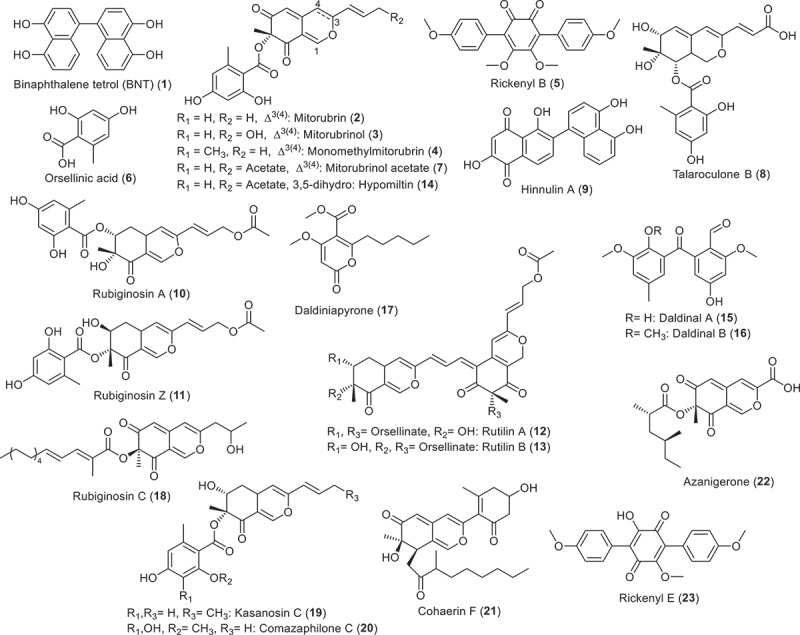
Stromatal chemotaxonomic marker compounds and other secondary metabolites from *Hypoxylon* spp. (Hashimoto et al. 1994; Stadler et al. 2004; Quang et al. 2005; Li et al. 2015; Shao et al. 2020).

The genus *Hypoxylon* remains one of the most widespread and largest in the Xylariales, even after various taxonomic changes that recently occurred (Lambert et al. 2019; Cedeño-Sanchez et al. 2023). For instance, the families of stromatic Xylariales have been segregated by Wendt et al. (2018), and *Hypoxylon* and its allies are now accommodated in their own family, Hypoxylaceae, based on a multi-locus genealogy that is partially supported by phylogenomics (cf. Wibberg et al. 2021). The species concept of the genus *Hypoxylon* and others in the Hypoxylaceae has also undergone drastic changes throughout the last decades. It is now based on a combination of holomorphic morphology and molecular phylogeny, aided by chemotaxonomic evidence (Kuhnert et al. 2014a, 2014b, 2015, 2017b; Sir et al. 2015; Lambert et al. 2019, 2021; Cedeño-Sanchez et al. 2023). One of the reasons for this change in paradigm is that these fungi possess an extraordinarily well-developed secondary metabolism (Helaly et al. 2018; Becker and Stadler 2021); see Figure 1 for some representative compounds. Some of these compounds constitute significant additional phenotypic traits that strongly correlate with their morphology and molecular phylogeny (Sir et al. 2019; Lambert et al. 2019, 2021).

Many ancient type specimens of *Hypoxylon* can neither be studied for anamorphic characters, nor sequenced. However, many old type specimens that were originally collected in the 18th century have retained their typical pigments (Stadler et al. 2008). Moreover, even the secondary metabolites of fossilised stromata of *H. fragiforme* were reported to remain stable even over long periods of time (Surup et al. 2018). Since the HPLC-based metabolite profiles were often found characteristic for a certain species, which is reflected by the colours of stromatal pigments in 10% KOH, chemotaxonomy can often aid in the discrimination of species within complexes of morphologically similar taxa. Nevertheless, modern taxonomic concepts require the availability of cultures, which can be subjected to DNA sequencing. Since the rDNA in Hypoxylaceae has proven rather unreliable, due to intragenomic polymorphisms on the one hand and redundancies on the other (cf. Cedeño-Sanchez et al. 2024), protein-coding genes that cannot be easily obtained from stromata has been favoured over ITS and LSU sequences since the advent of molecular phylogeny (Hsieh et al. 2005).

Some more recent reports on new taxa described from the Neotropics focused on describing and delineating novel species based on teleomorphic morphology (see, for example, Fournier 2014; Fournier et al. 2014, 2015; Fournier and Lechat 2015). These studies included detailed morphological descriptions and excellent macro/microscopic images, but it is unfortunate that they were not accompanied by molecular data. In particular, we now know that the tropical species of the genus *Hypoxylon* cannot easily be segregated without careful assessment of both their morphological and molecular traits. In addition, molecular and chemotaxonomic data repeatedly shed more light on the ecology of the respective fungi. For instance, it has been possible to elucidate the life cycle of the cosmopolitan tropical nodulisporic acid producing endophytes based on a comparison of secondary metabolite profiles and DNA sequences of an ascospore-derived culture (Bills et al. 2012). This resulted in the description of *H. pulicicidum*. In other cases, it may be difficult to determine whether a certain species that was recently reported from Asia without morphological studies of the type material is really different from the original one that was described from Europe or the Neotropics, even if extensive molecular data have been reported from the Asian material.

We here report on a re-assessment of previously studied Neotropical specimen that were erected by highly capable French Citizen Scientists in the past decade. We hope that we can thereby contribute to the knowledge on the taxonomy of *Hypoxylon* and aid other scientists to identify these taxa more easily in the future. We have also encountered a specimen in the USA that we regard as suitable to represent the epitype of the important taxon *Sphaeria perforata.*

## 2. Materials and methods

### 2.1. Sample sources

All scientific names of fungi are given without authorities or publication details and follow the entries in Index Fungorum (http://www.indexfungorum.org). Type and reference specimens were provided by the Université de Lille herbarium (LIP). Fungal cultures were provided from the Belgian Coordinated Collections of Microorganisms (MUCL).

### 2.2. Molecular phylogenetic analyses

The taxa for this study were selected according to recent, representative studies and are listed in Table 1. DNA extraction and amplification for the newly included strains were performed in accordance with Cedeño-Sanchez et al. (2023).

**Table 1.** Strains used in the phylogenetic analyses, including the strain IDs, GenBank accession numbers, and the references where the sequence data have been originally generated.

Species	Strain number	GenBank accession number				Origin	References
		ITS	LSU	*RPB2*	*TUB2*		
*Annulo hypoxylon annulatum*	CBS 140775	KY610418	KY610418	KY624263	KX376353	USA (ET)	Kuhnert et al. (2017a; *TUB2*), Wendt et al. (2018; ITS, LSU, *RPB2*)
*A. michelianum*	CBS 119993	KX376320	KY610423	KY624234	KX271239	Spain	Kuhnert et al. (2017a; ITS, *TUB2*), Wendt et al. (2018; LSU, *RPB2*)
*A. truncatum*	CBS 140778	KY610419	KY610419	KY624277	KX376352	USA (ET)	Kuhnert et al. (2017a; *TUB2*), Wendt et al. (2018; ITS, LSU, *RPB2*)
*Daldinia bambusicola*	CBS 122872	KY610385	KY610431	KY624241	AY951688	Thailand (T)	Hsieh et al. (2005; *TUB2*), Wendt et al. (2018; ITS, LSU, *RPB2*)
*D. childiae*	CBS 122881	KU683757	MH874773	KU684290	KU684129	France (T)	U’Ren et al. (2016; ITS, *TUB2*, *RPB2*), Vu et al. (2019; LSU)
*D. concentrica*	CBS 113277	AY616683	KY610434	KY624243	KC977274	Germany	Triebel et al. (2005; ITS), Kuhnert et al. (2014a; *TUB2*), Wendt et al. (2018; LSU, *RPB2*)
*D. dennisii*	CBS 114741	JX658477	KY610435	KY624244	KC977262	Australia (T)	Stadler et al. (2014; ITS), Kuhnert et al. (2014a; *TUB2*), Wendt et al. (2018; LSU, *RPB2*)
*D. eschscholtzii*	MUCL 45435	JX658484	KY610437	KY624246	KC977266	Benin	Stadler et al. (2014; ITS), Kuhnert et al. (2014a; *TUB2*), Wendt et al. (2018; LSU, *RPB2*)
*D. petriniae*	MUCL 49214	AM749937	KY610439	KY624248	KC977261	Austria (ET)	Bitzer et al. (2008; ITS), Kuhnert et al. (2014a; *TUB2*), Wendt et al. (2018; LSU, *RPB2*)
*D. placentiformis*	MUCL 47603	AM749921	KY610440	KY624249	KC977278	Mexico	Stadler et al. (2014; ITS), Kuhnert et al. (2014a; *TUB2*), Wendt et al. (2018; LSU, *RPB2*)
*D. vernicosa*	CBS 119316	KY610395	KY610442	KY624252	KC977260	Germany (ET)	Kuhnert et al. (2014b; *TUB2*), Wendt et al. (2018; ITS, LSU, *RPB2*)
*Durotheca comedens*	YMJ 90071615	EF026128		JX507793	EF025613	Taiwan of China (T)	Ju et al. (2003) as *Theissenia*
*Du. crateriformis*	GMBC 0205	MH645426	MH645425	MH645427	MH049441	China (T)	de Long et al. (2019)
*Du. guizhouensis*	GMBC 0065	MH645423	MH645421	MH645422	MH049439	China (T)	de Long et al. (2019)
*Du. rogersii*	YMJ 92031201	EF026127		JX507794	EF025612	Taiwan of China	Ju et al. (2007) as *Theissenia*
*D. rogersii*	GMBC 0204	MH645433	MH645434	MH645435	MH049449	China	de Long et al. (2019)
*Graphostroma platystomum*	CBS 27087	JX658535	DQ836906	KY624296	HG934108	France (T)	Zhang et al. (2006; LSU), Stadler et al. (2014; ITS), Koukol et al. (2015; *TUB2*), Wendt et al. (2018; *RPB2*)
*Hypomontagnella barbarensis*	STMA 14081	MK131720	MK131718	MK135891	MK135893	Argentina (T)	Lambert et al. (2019)
*Hyp. monticulosa*	MUCL 54604	KY610404	KY610487	KY624305	KX271273	French Guiana	Wendt et al. (2018)
*Hyp. submonticulosa*	CBS 115280	KC968923	KY610457	KY624226	KC977267	France	Kuhnert et al. (2014a; ITS, *TUB2*), Wendt et al. (2018; LSU, *RPB2*)
*Hypoxylon addis*	MUCL 52797	KC968931	ON954141	OP251037	KC977287	Ethiopia (T)	Kuhnert et al. (2014a; ITS, *TUB2*), Cedeño-Sanchez et al. (2023; LSU, *RPB2*)
*H. aveirense*	MUM 1940	MN053021	ON954142	OP251028	MN066636	Portugal (T)	Vicente et al. (2021; ITS, *TUB2*), Cedeño-Sanchez et al. (2023; LSU, *RPB2*)
*H. baruense*	DSM 115549	MN056428	ON954143	PP732079	MK908142	Panama (T)	Cedeño-Sanchez et al. (2020; ITS, *TUB2*), Cedeño-Sanchez et al. (2023; LSU), *RPB2* This study
*H. canariense*	MUCL 47224	ON792787	ON954140	OP251029	ON813073	Spain, Canary Islands (PT)	Cedeño-Sanchez et al. (2023)
*H. carneum*	MUCL 54177	KY610400	KY610480	KY624297	KX271270	France	Wendt et al. (2018)
*H. cercidicola*	CBS 119009	KC968908	KY610444	KY624254	KC977263	France	Kuhnert et al. (2014a; ITS, *TUB2*), Wendt et al. (2018; LSU, *RPB2*)
*H. chionostomum*	STMA 14060	KU604563	ON954144	OP251030	ON813072	Argentina	Sir et al. (2016; ITS); Cedeño-Sanchez et al. (2023)
*H. chrysalidosporum*	FCATAS 2710	OL467294	OL615106	OL584222	OL584229	China (T)	Ma et al. (2022)
*H. crocopeplum*	CBS 119004	KC968907	KY610445	KY624255	KC977268	France	Kuhnert et al. (2014a; ITS, *TUB2*), Wendt et al. (2018; LSU, *RPB2*)
*H. cyclobalanopsidis*	FCATAS 2714	OL467298	OL615108	OL584225	OL584232	China (T)	Ma et al. (2022)
*H. damuense*	FCATAS 4207	ON075427	ON075433	ON093251	ON093245	China (T)	Song et al. (2022)
*H. diperithecium*	FCATAS 4226	ON178671	ON350864	ON365561	ON365565	China (T)	Zhu et al. (2023)
*H. duranii*	ATCC 58730	PP718984	PP729636	PP732085	PP721316	Mexico (T)	This study
*H. dussii*	MUCL 53766	PP718981	PP729635	PP732081	PP721315	Guadeloupe(T)	This study
*H. erythrostroma*	MUCL 53759	KC968910	ON954154	OP251031	KC977296	Martinique	Kuhnert et al. (2014a; ITS2, *TUB2*), Cedeño-Sanchez et al. (2023)
*H. eurasiaticum*	MUCL 57720	MW367851		MW373852	MW373861	Iran (T)	Lambert et al. (2021)
*H. fendleri*	MUCL 54792	KF234421	KY610481	KY624298	KF300547	French Guiana	Kuhnert et al. (2014a; ITS, *TUB2*), Wendt et al. (2018; LSU, *RPB2*)
*H. ferrugineum*	CBS 141259	KX090079			KX090080	Austria	Friebes and Wendelin (2016)
*H. fragiforme*	MUCL 51264	KC477229	KM186295	MK887342	KX271282	Germany (ET)	Stadler et al. (2013; ITS), Daranagama et al. (2015; LSU, *RPB2*), Wendt et al. (2018; *TUB2*)
*H. fuscoides*	MUCL 52670	ON792789	ON954145	OP251038	ON813076	France (T)	Cedeño-Sanchez et al. (2023) (Species described by Fournier et al. 2010)
*H. fuscum*	CBS 113049	KY610401	KY610482	KY624299	KX271271	Germany (ET)	Wendt et al. (2018)
*H. gibriacense*	MUCL 52698	KC968930	ON954146	OP251026	ON813074	France (T)	Kuhnert et al. (2014a; ITS), Cedeño-Sanchez et al. (2023; LSU, *TUB2, RPB2*)
*H. griseobrunneum*	CBS 33173	KY610402	KY610483	KY624300	KC977303	India (T)	Kuhnert et al. (2014a; *TUB2*), Wendt et al. (2018; ITS, LSU, *RPB2*)
*H. guilanense*	MUCL 57726	MT214997	MT214992	MT212235	MT212239	Iran (T)	Pourmoghaddam et al. (2020)
*H. haematostroma*	MUCL 53301	KC968911	KY610484	KY624301	KC977291	Martinique (ET)	Wendt et al. (2018; LSU, *RPB2*), Kuhnert et al. (2014a; ITS, *TUB2*)
*H. hainanense*	FCATA S2712	OL467296	OL616132	OL584224	OL584231	China (T)	Ma et al. (2022)
*H. hinnuleum*	ATCC 36255, MUCL 3621	MK287537	MK287549	MK287562	MK287575	USA (T)	Sir et al. (2019)
*H. howeanum*	MUCL 47599	AM749928	KY610448	KY624258	KC977277	Germany	Bitzer et al. (2008; ITS), Kuhnert et al. (2014a; *TUB2*), Wendt et al. (2018; LSU, *RPB2*)
*H. hypomiltum*	MUCL 51845	KY610403	KY610449	KY624302	KX271249	Guadeloupe	Wendt et al. (2018)
*H. invadens*	MUCL 51475	MT809133	MT809132	MT813037	MT813038	France (T)	Becker et al. (2020)
*H. investiens*	CBS 118183	KC968925	KY610450	KY624259	KC977270	Malaysia	Kuhnert et al. (2014a; ITS, *TUB2*), Wendt et al. (2018; LSU, *RPB2*)
*H. isabellinum*	MUCL 53308	KC968935	ON954155	OP251032	KC977295	Martinique (T)	Kuhnert et al. (2014a; ITS, *TUB2*), Cedeño-Sanchez et al. (2023)
*H. laschii*	MUCL 52796	JX658525	ON954147	OP251027	ON813075	France	Stadler et al. (2014; ITS), Cedeño-Sanchez et al. (2023; LSU, *TUB2, RPB2*)
*H. lateripigmentum*	MUCL 53304	KC968933	KY610486	KY624304	KC977290	Martinique (T)	Kuhnert et al. (2014a; ITS, *TUB2*), Wendt et al. (2018; LSU, *RPB2*)
*H. lechatii*	MUCL 54609	KF923407	ON954148	OP251033	KF923405	French Guiana	Kuhnert et al. (2014b; ITS, *TUB2*), Cedeño-Sanchez et al. (2023; LSU, *RPB2*)
*H. lenormandii*	CBS 119003	KC968943	KY610452	KY624261	KC977273	Ecuador	Kuhnert et al. (2014b; ITS, *TUB2*), Wendt et al. (2018; LSU, *RPB2*)
*H. lienhwacheense*	MFLUCC 14-1231	KU604558	MK287550	MK287563	KU159522	Thailand	Sir et al. (2016; ITS, *TUB2*), Sir et al. (2019; LSU, *RPB2*)
*H. lividipigmentum*	STMA14045	ON792788	ON954149	PP732080	ON813077	Argentina	Cedeño-Sanchez et al. (2023; ITS, LSU, *TUB2*), *RPB2* this study
*H. lividipigmentum*	BCRC 34077	JN979433			AY951735	Mexico (IT)	Hsieh et al. (2005)
*H. macrocarpum*	CBS 119012	ON792785	ON954151	OP251034	ON813071	Germany	Cedeño-Sanchez et al. (2023)
*H. medogense*	FCATAS 4061	ON075425	ON075431	ON093249	ON093243	China (T)	Song et al. (2022)
*H. munkii*	MUCL 53315	KC968912	ON954153	OP251035	KC977294	Martinique	Kuhnert et al. (2014a; ITS, *TUB2*), Cedeño-Sanchez et al. (2023)
*H. musceum*	MUCL 53765	KC968926	KY610488	KY624306	KC977280	Guadeloupe	Kuhnert et al. (2014a; ITS, *TUB2*), Wendt et al. (2018; LSU, *RPB2*)
*H. ochraceum*	MUCL 54625	KC968937		KY624271	KC977300	Martinique (ET)	Kuhnert et al. (2014a; ITS, *TUB2*), Wendt et al. (2018; *RPB2*)
*H. olivaceopigmentum*	DSM 107924	MK287530	MK287542	MK287555	MK287568	USA (T)	Sir et al. (2019)
*H. perforatum*	CBS 115281	KY610391	KY610455	KY624224	KX271250	France	Wendt et al. (2018)
*H. perforatum*	STMA 23134	PP718982	PP729634	PP732084	PP721314	USA (ET)	This study
*H. petriniae*	CBS 114746	KY610405	KY610491	KY624279	KX271274	France (T)	Wendt et al. (2018)
*H. pilgerianum*	STMA 13455	KY610412	KY610412	KY624308	KY624315	Martinique	Wendt et al. (2018)
*H. porphyreum*	CBS 119022	KC968921	KY610456	KY624225	KC977264	France	Kuhnert et al. (2014a; ITS, *TUB2*), Wendt et al. (2018; LSU, *RPB2*)
*H. pseudofuscum*	DSM 112038	MW367857	MW367848	MW373858	MW373867	Germany (T)	Lambert et al. (2021)
*H. pulicicidum*	CBS 122622	JX183075	KY610492	KY624280	JX183072	Martinique (T)	Bills et al. (2012; ITS, *TUB2*), Wendt et al. (2018; LSU, *RPB2*)
*H. rickii*	MUCL 53309	KC968932	KY610416	KY624281	KC977288	Martinique (ET)	Kuhnert et al. (2014a; ITS, *TUB2*), Wendt et al. (2018; LSU, *RPB2*)
*H. rubiginosum*	MUCL 52887	KC477232	KY610469	KY624266	KY624311	Germany (ET)	Stadler et al. (2013; ITS), Wendt et al. (2018; *TUB2*, LSU, *RPB2*)
*H. samuelsii*	MUCL 51843	KC968916	KY610466	KY624269	KC977286	Guadeloupe (ET)	Kuhnert et al. (2014a; ITS, *TUB2*), Wendt et al. (2018; LSU, *RPB2*)
*H. sofainense*	MUCL 54170	PP718983	PP729633	PP732083	PP721313	Guadeloupe (T)	This study
*H. sporistriatatunicum*	DSM 115550	MN056426	ON954150	OP251036	MK908140	Panama (T)	Cedeño-Sanchez et al. (2020; ITS, *TUB2*), Cedeño-Sanchez et al. (2023; LSU, *RPB2*)
*H. subticinense*	MUCL 53752	KC968913	ON954152	PP732082	KC977297	French Guiana	Kuhnert et al. (2014b; ITS, *TUB2*), Cedeño-Sanchez et al. (2023; LSU), *RPB2* this study
*H. subgilvum*	STMA 24034	PP718985	PP729637	PP732082	PP721317	Panama	This study
*H. texense*	DSM 107933	MK287536	MK287548	MK287561	MK287574	USA (T)	Sir et al. (2019)
*H. tibeticum*	FCATAS 4022	OR654146	OR654303	ON254302	ON230084	China (T)	Zhu et al. (2023)
*H. ticinense*	CBS 115271	JQ009317	KY610471	KY624272	AY951757	France	Hsieh et al. (2005; ITS, *TUB2*), Wendt et al. (2018; LSU, *RPB2*)
*H. trugodes*	MUCL 54794	KF234422	KY610493	KY624282	KF300548	Sri Lanka (ET)	Kuhnert et al. (2014a; ITS, *TUB2*), Wendt et al. (2018; LSU, *RPB2*)
*H. vogesiacum*	CBS 115273	KC968920	KY610417	KY624283	KX271275	France	Kuhnert et al. (2014a; ITS), Kuhnert et al. (2017a; *TUB2*), Wendt et al. (2018; LSU, *RPB2*)
*H. wuzhishanense*	FCATAS 2708	OL467292	OL615104	OL584220	OL584227	China (T)	Ma et al. (2022)
*H. zangii*	FCATAS 4029	ON075423	ON075429	ON093247	ON093241	China (T)	Song et al. (2022)
*Jackrogersella cohaerens*	CBS 119126	KY610396	KY610497	KY624270	KY624314	Germany	Wendt et al. (2018)
*J. multiformis*	CBS 119016	KC477234	KY610473	KY624290	KX271262	Germany (ET)	Kuhnert et al. (2014a; ITS), Kuhnert et al. (2017a; *TUB2*), Wendt et al. (2018; LSU, *RPB2*)
*Parahypoxylon papillatum*	ATCC 58729	KC968919	KY610454	KY624223	KC977258	USA (T)	Kuhnert et al. (2014a; ITS, *TUB2*), Wendt et al. (2018; LSU, *RPB2*)
*Pa. ruwenzoriense*	MUCL 51392	ON792786	ON954156	OP251039	ON813078	D. R. Congo (T)	Cedeño-Sanchez et al. (2023)
*Pyrenopolyporus hunteri*	MUCL 52673	KY610421	KY610472	KY624309	KU159530	Ivory Coast (ET)	Kuhnert et al. (2017; *TUB2*), Wendt et al. (2018; ITS, LSU, *RPB2*)
*Py. laminosus*	MUCL 53305	KC968934	KY610485	KY624303	KC977292	Martinique (T)	Kuhnert et al. (2014a; ITS, *TUB2*), Wendt et al. (2018; LSU, *RPB2*)
*Py. nicaraguense*	CBS 117739	AM749922	KY610489	KY624307	KC977272	Burkina Faso	Bitzer et al. (2008; ITS), Kuhnert et al. (2014a; *TUB2*), Wendt et al. (2018; LSU, *RPB2*)
*Rhopalostroma angolense*	CBS 126414	KY610420	KY610459	KY624228	KX271277	Ivory Coast	Wendt et al. (2018)
*Rostrohypoxylon terebratum*	CBS 119137	DQ631943	DQ840069	DQ631954	DQ840097	Thailand (T)	Fournier et al. (2010)
*Ruwenzoria pseudoannulata*	MUCL 51394	KY610406	KY610494	KY624286	KX271278	D. R. Congo (T)	Wendt et al. (2018)
*Thamnomyces dendroidea*	CBS 123578	FN428831	KY610467	KY624232	KY624313	French Guiana (T)	Stadler et al. (2010; ITS), Wendt et al. (2018; *TUB2*, LSU, *RPB2*)
*Xylaria arbuscula*	CBS 126415	KY610394	KY610463	KY624287	KX271257	Germany	Fournier et al. (2011; ITS), Wendt et al. (2018; *TUB2*, LSU, *RPB2*)
*X. hypoxylon*	CBS 122620	KY610407	KY610495	KY624231	KX271279	Sweden (ET)	Wendt et al. (2018)

Type specimens are labelled with T (holotype), IT (isotype), PT (paratype), and ET (epitype).

Sequences were analysed and processed in Geneious prime 2023 (https://www.geneious.com). The generated sequence data were complemented by available sequence data from GenBank and the data sets for each genetic marker aligned using MAFFT online (http://mafft.cbrc.jp/alignment/server/., Katoh et al. 2019), and manually curated in Geneious prime 2023 (https://www.geneious.com). A maximum-likelihood phylogenetic tree was constructed using IQ-TREE v. 2.1.3 [-b 1000 -abayes -m MFP -nt AUTO] (Minh et al. 2020). The appropriate nucleotide exchange model was selected by ModelFinder (Chernomor et al. 2016; Kalyaanamoorthy et al. 2017) based on Bayesian inference criterion. Branch support was calculated using non-parametric bootstrap (Felsenstein 1985), and approximate Bayes test (Anisimova et al. 2011).

### 2.3. HPLC profiling

Stromatal extracts were obtained by removing small amounts of the surface and perithecial layer (approximately 1 mm^3^), transferring them to 1.5 mL reaction tubes and adding 100 µL of methanol. The material was extracted for 15 min at 40 °C in an ultrasonic bath. The tubes were then centrifuged, and the supernatant was transferred to a new tube and used for chemical analyses using high-performance liquid chromatography coupled to a diode array detector and an Electrospray mass spectrometer (HPLC/DAD-ESI-MS). The instrumental settings and conditions were as described by Lambert et al. (2021).

## 3. Results

### 3.1. Phylogenetic analysis

A molecular phylogeny was inferred from four different loci (ITS, LSU, *RPB2*, and *TUB2*), representing in total 96 species. The final data matrix comprised 388 sequences (23 generated in this study, and complemented by sequences available from GenBank, NCBI). The MAFFT alignments consisted of 2,031 positions for the ITS; 5,376 for the LSU; 2,564 for the *TUB2* and 4,091 for *RPB2*. Each alignment is available in the supplementary (SI Tables 3–6). Sequences of representative genera of the Hypoxylaceae were included: *Annulohypoxylon* (3 strains), *Daldinia* (8 strains), *Durotheca* (5 strains), *Hypomontagnella* (3 strains), *Hypoxylon* (63 strains), *Jackrogersella* (2 strains), *Parahypoxylon* (2 strains), *Pyrenopolyporus* (3 strains), as well as *Rhopalostroma*, *Rostrohypoxylon*, *Ruwenzoria* and *Thamnomyces* (1 strain each). Three members of Xylariaceae and Graphostromataceae (*Xylaria hypoxylon, X. arbuscula*, and *Graphostroma platystomum*) served as outgroup.

**Figure 2.**
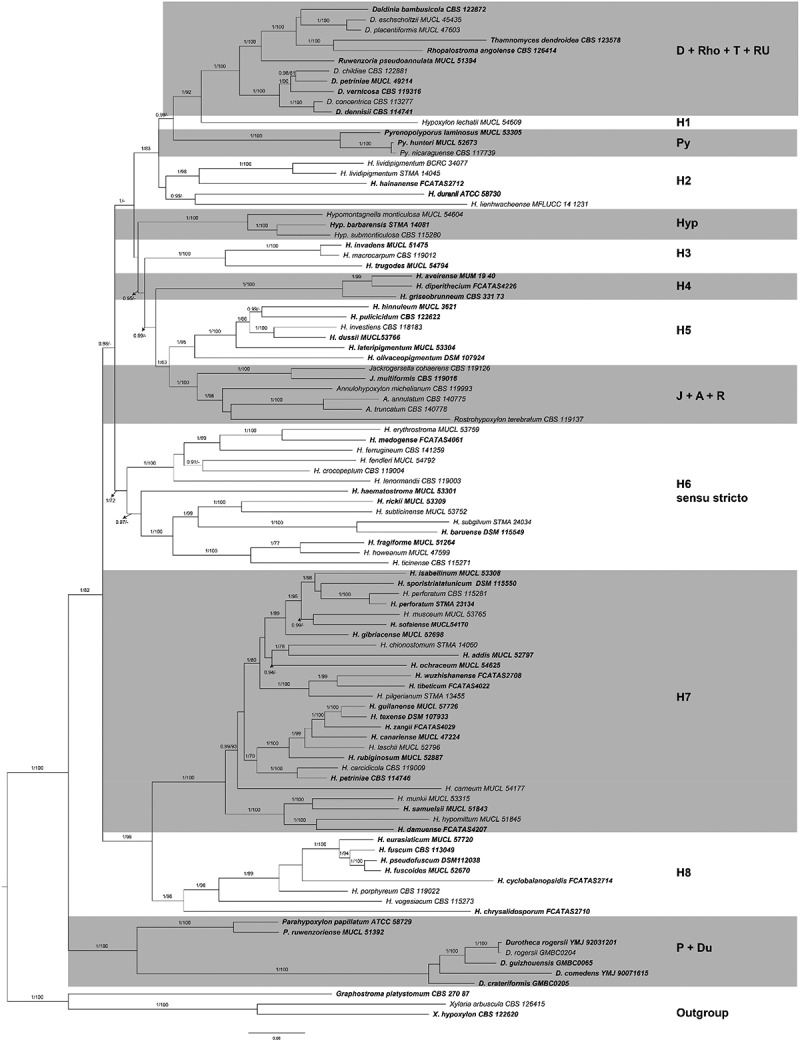
Inferred molecular phylogenetic maximum likelihood (lLn = −124,622.0188) tree of selected Hypoxylaceae, Graphostromataceae and Xylariaceae calculated by using IQ-Tree with posterior probability support calculated from Bayesian inference methodology and support values generated from 1,000 bootstrap replicates using a multigene alignment (ITS, LSU, *TUB2*, and *RPB2*). The tree was rooted with *Xylaria hypoxylon* CBS 122620, *X. arbuscula* CBS 126415 (Xylariaceae), and *Graphostroma platystomum* CBS 270.87 (Graphostromataceae). Type material is highlighted in **bold** letters. Bayesian posterior probability scores ≥ 0.95/Bootstrap support values ≥ 70 are indicated along branches.

The species *H. dussii* and *H. sofaiense* were described by Fournier et al. (2015) and sequenced for the first time in this study. *Hypoxylon dussii* clustered in a clade containing *H.* *investiens*, *H*. *pulicicidum*, *H.* *hinnuleum*, and *H. lateripigmentum* with strong support (1/100) in clade H5. On the other hand, *H. sofaiense* clustered with *H.* *musceum*, *H.* *perforatum*, *H.* *isabellinum*, and *H. sporistriatatunicum* with high support (1/95) in clade H7 (Figure 2). The close phylogenetic relationship of *H. dussii* with *H. investiens*; and that of *H. sofaiense* with different species of the *H. perforatum* clade H7 is in full accordance with the morphological similarities previously noted by Fournier et al. (2015).

### 3.2. Taxonomy

#### 3.2.1. Epitypification

***Hypoxylon perforatum*** (Schwein.) Fr., Summa veg. Scand., Post Section. (Stockholm): 384 (1849)

**Basionym**: *Sphaeria perforata* Schwein., Naturf. Ges. Leipzig 1: 31. 1822; Pers., Syst. Mycol. II, p. 340. 1823., nom. sanct.

**Synonyms** (fide Ju and Rogers 1996, supported by our own observations on most of the type specimens):

≡ *Sphaeria durissima* Schwein., Naturf. Ges. Leipzig 1: 32. 1822.

= *Sphaeria decorticata* Schwein., Trans. Amer. Philos. Soc., n. ser., 4: 191. 1832.

= *Sphaeria catalpae* Schwein., Trans. Amer. Philos. Soc., n. ser., 4: 193. 1832.

≡ *Hypoxylon decorticatum* (Schwein.) M. A. Curtis, Geol. Nat. Hist. Surv. North Carolina, pt. III, p. 140. 1867.

= *Hypoxylon luridum* Nitschke, Pyrene. Germ., p. 31. 1867.

≡ *Hypoxylon catalpae* (Schwein.) Sacc., Syll. Fung. I, p. 392. 1882.

≡ *Hypoxylon leucostigma* (Lév.) Sacc., Syll. Fung. I, p. 367. 1882.

= *Hypoxylon plumbeum* Speg., Anales Soc. Ci. Argent. 18: 270. 1884.

= *Hypoxylon rubiginosum* (Pers) Fr. var. *microcarpum* Speg., Anales Mus. After. Hist. Nat. Buenos Aires 17: 120. 1908.

≡ *Hypoxylon rubiginosum* (Pers.) Fr. var. *perforatum* (Pers.: Fr.) L. E. Petrini apud L. E. Petrini & Müller, Mycol. Helv. 1: 531. 1986.

**Lectotype**: USA, North Carolina, Salem and Pennsylvania, Bethlehem, Syn. 1194, corticated wood (PH, Lectotype [selected by Ju and Rogers (1996)] of *Sphaeria perforata*).

**Epitype**: Designated here, USA, North Carolina, Highlands, Blue Valley Campground, on bark, 16 Aug. 2023 (ex-type culture STMA 23134, deposited with DSMZ). GenBank Acc. No.: PP718982 (ITS), PP729634 (LSU), PP732084 (*RPB2*), PP721314 (*TUB2*).

**Figure 3.**
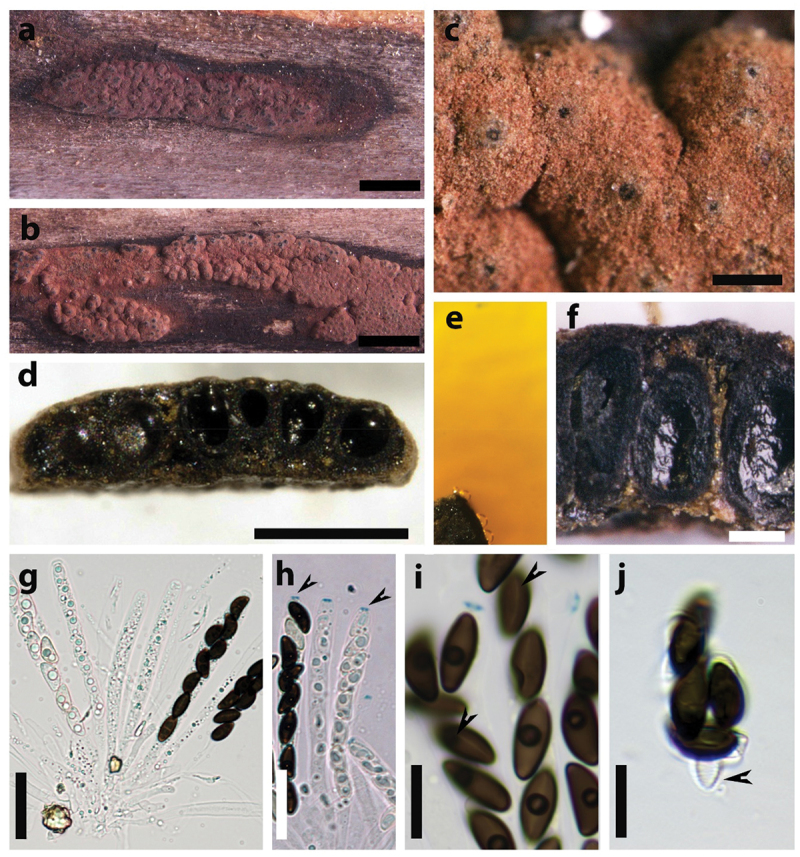
*Hypoxylon perforatum*. Epitype specimen (a – b) Stromata on substrate. (c) Ostioles. (d) Vertical section of stroma showing perithecia. (e) KOH extractable pigments. (f) Detail of granules between perithecia. (g) Asci in water. (h) Amyloid apical apparatus. (i) Ascospore with straight germ slit. (j) Dehiscent perispore in 10% KOH and slightly striated epispore. Scale bars: a – b = 0.1 cm; c = 0.2 mm; d = 0.3 mm; f = 0.1 mm; g – h = 25 µm; i – j =10 µm.

**Notes**: This species belongs to the *H. rubiginosum* complex and its teleomorph is well-characterised by stromatal KOH – extractable pigments with light greenish colour (owing to the presence of hypomiltin, rather than the orange mitorubrin derivatives that are prevailing in many other species of this complex; cf. Hellwig et al. 2005) and the size of the ascospores: 10–12.2 × 3.6–4.7 µm (*n* = 15) and the ascus: 77.8–95.4 × 6.4–7.8 µm (*n* = 8). Notably, the specimen designated here is perfectly suitable to serve as epitype due to its occurrence from the same geographic area as the holotype. Actually, the type material of *S. perforata* was studied by Stadler et al. (2008) and found to contain hypomiltin derivatives and orsellinic acid as well.

**MBT no**: 10019968 (Figure 3).

### 3.2.2. Chemotaxonomic part

In total, 28 specimens of *Hypoxylon*, grouped in 16 species, collected from the Neotropics between 2010 and 2014 were assessed for chemotaxonomic markers in their stromatal crude extracts using HPLC-ESI-MS. This information is complementary to the morphological description and emends their placement in the Hypoxylaceae in the absence of cultures for molecular studies. Nevertheless, we were able to retrieve pure ex-type cultures for *H. dussii* and *H. sofaiense* that were unfortunately not reported in the respective protologues. Those cultures were sequenced and included in the updated phylogenetic tree presented above.

Deviating from the customary mode that has traditionally been applied in our previous papers, we first list the results of the chemotaxonomic studies and then comment on the results in the discussions. We wish to point out that our results were not always based on the availability of standards, and even in those cases where standards were available from previous preparative studies, the stereochemistry and identity of the compounds is not 100% sure because HPLC-MS-based methods cannot reveal the absolute chemical structures.

***Hypoxylon dussii*** J. Fourn. & Lechat, Ascomycete.org 7(5): 161 (2015)

**Secondary metabolites**: Stromata contain binaphthalene tetrol (**1**, BNT), mitorubrin (**2**), mitorubrinol (**3**) (Hellwig et al. 2005; Stadler et al. 2007) and two unknown isomeric compound (**UC1**/**2**: T_R_ 5.03/5.11 min, UV λ_max_: 229, 339 nm, M._f._ C_22_H_22_O_8_, M._wt._ 414) together with four other components (**UC3**: T_R_ 2.07 min, UV λ_max_: 227, 336 nm, M._f._ C_17_H_16_O_6_, M._wt._ 316; **UC4**: T_R_ 3.13 min, UV λ_max_: 224, 336 nm, M._f._ C_22_H_22_O_8_, M._wt._ 414; **UC5**: T_R_ 3.83 min, UV λ_max_: 224, 335 nm, M._f._ C_22_H_24_O_10_, M._wt._ 448) as major components as well as monomethylmitorubrin (**4**) (Yamazaki et al. 2010) and rickenyl B (**5**) (Kuhnert et al. 2015) as minor components (SI Figure S1).

**Material examined**: French West Indies, Guadeloupe, Sainte-Rose, Sofaïa, path to Saut des Trois Cornes, mesophilic rainforest, on dead corticated branch, Nov. 2005, leg. C. Lechat, CLL 5439 (Holotype LIP; ex-holotype culture MUCL 53766). French West Indies, Guadeloupe, Sainte-Rose, Sofaïa, path to Saut des Trois Cornes, mesophilic forest, on dead corticated branch, Nov. 2005, leg. C. Lechat, CLL 5433 (Paratype LIP).

***Hypoxylon sofaiense*** J. Fourn. & Lechat, Ascomycete.org 7(5): 202 (2015)

**Secondary metabolites**: Stromata contain orsellinic acid (**6**) and mitorubrinol acetate (**7**) (Hellwig et al. 2005; Stadler et al. 2007) as a major component as well as talaroculone B (**8**) (Ren et al. 2017) as a minor component (SI Figure S2).

**Material examined**: French West Indies, Guadeloupe, Sainte-Rose, Sofaïa, path to Saut des Trois Cornes, mesophilic rainforest, on dead corticated branch ca. 2 cm diam., Nov. 2005, leg. C. Lechat, CLL 5406 (Holotype LIP; ex-holotype culture MUCL 54170).

***Hypoxylon cypraeisporum*** J. Fourn. & Lechat, Ascomycete.org 7(1): 10 (2015)

**Secondary metabolites**: Stromata contain hinnulin A (**9**) (Kuhnert et al. 2017b) as a major component (SI Figure S3).

**Material examined**: French West Indies, Guadeloupe, Sainte-Rose, Trace de Sofaïa, chemin du Saut des Trois Cornes, rainforest, on corticated branch, Nov. 2005, leg. C. Lechat, CLL 5425 (Holotype LIP). French West Indies, Guadeloupe, Aug. 2004, on a corticated twig, associated with *Xylaria boergesenii*, leg. C. Lechat, CLL 2239 (Paratype LIP). French West Indies, Guadeloupe, Sainte-Rose, rivière Janikeste, on bark, associated with *Xylaria boergesenii*, Aug. 2008, leg. C. Lechat, CLL 8227 (Paratype LIP).

***Hypoxylon nudum*** J. Fourn. & Lechat, Ascomycete.org 7(2): 80 (2015)

**Secondary metabolites**: The stromatal crude extracts did not reveal any prominent, known secondary metabolites.

**Material examined**: French Guiana, Régina, Nouragues natural reserve, Inselberg camp, trail to Pararé, ca. 1 km from the camp, rainforest, on bark of unidentified dead standing trunk, Jun. 2012, leg. J. Fournier, GYJF 12134 (Holotype LIP).

***Hypoxylon verruciperisporium*** J. Fourn. & Lechat, Ascomycete.org 7(2): 91 (2015)

**Secondary metabolites**: Stromata contain mitorubrinol acetate (**7**), rubiginosins A (**10**)/Z (**11**) (Hellwig et al. 2005; Quang et al. 2005; Stadler et al. 2007; Becker et al. 2021) and rutilins A (**12**)/B (**13**) (Quang et al. 2005) as major components alongside with orsellinic acid (**6**) and hypomiltin (**14**) (Hellwig et al. 2005; Stadler et al. 2007) as minor metabolites (SI Figure S4).

**Material examined**: French Guiana, Sinnamary, Paracou, CIRAD field station, lowland rainforest, dead corticated branch, Jun. 2012, leg. J. Fournier, GYJF 12239 (Holotype LIP). French Guiana, Régina, Nouragues natural reserve, Inselberg camp, rainforest, dead corticated branch, Jun. 2012, leg. J. Fournier & C. Lechat, GYJF 12110 (Paratype LIP).

***Hypoxylon arawakianum*** J. Fourn. & Lechat, Ascomycete.org 7(5): 149 (2015)

**Secondary metabolites**: Stromata contain four unknown compounds (**UC1**: T_R_ 7.60 min, UV λ_max_: 198, 220, 307 nm, M._f._ C_23_H_24_O_10_, M._wt._ 460; **UC2**: T_R_ 9.32 min, UV λ_max_: 199, 221, 306 nm, M._f._ C_26_H_40_O_14_, M._wt._ 576; **UC3**: T_R_ 10.09 min, UV λ_max_: 222, 306 nm, M._f._ C_30_H_46_O_16_, M._wt._ 662; **UC4**: T_R_ 10.18 min, UV λ_max_: 198, 223, 306 nm, M._f._ C_30_H_46_O_16_, M._wt._ 662) as major components (SI Figure S5).

**Material examined**: French West Indies, Martinique, Le Carbet, Anse Turin, path to Etin of lodge, on dead stems of *Ricinus communis* (Euphorbiaceae), Aug. 2013, leg. J. Fournier, MJF 13020 (Holotype LIP). French West Indies, Guadeloupe, Sainte-Rose, Sofaïa, path to Saut des Trois Cornes, mesophilic forest, on dead corticated wood, Sept. 2003, leg. C. Lechat, CLL 1039 (Paratype LIP).

***Hypoxylon subtrugodes*** J. Fourn. & Lechat, Ascomycete.org 7(5): 207 (2015)

**Secondary metabolites**: Stromata revealed BNT (**1**), daldinals A (**15**)/B (Hashimoto et al. 1994) (**16**) (Hellwig et al. 2005; Stadler et al. 2007) as major components with two unknown compounds (**UC1**: T_R_ 9.54 min, UV λ_max_: 227 nm, M._f._ C_23_H_28_O_9_, M._wt._ 448; **UC2**: T_R_ 11.38 min, UV λ_max_: 268 nm, M._f._ C_23_H_28_O_8_, M._wt._ 432) as minor components (SI Figure S6).

**Material examined**: French West Indies, Martinique, Case-Pilote, Fond Boucher, trail to Morne Venté, xero-to mesophilic forest, on dead corticated branch, Aug. 2013, leg. J. Fournier, MJF 13277 (Holotype LIP). French West Indies, Martinique, Case-Pilote, Fond Boucher, trail to Morne Venté, xero-to mesophilic forest, on dead corticated twig 6 mm diam, Aug. 2010, MJF 10119 (Paratype LIP).

***Hypoxylon leprieurianum*** J. Fourn. & Lechat, Ascomycete.org 7(2): 76 (2015)

**Secondary metabolites**: Stromata contain mitorubrinol (**3**), mitorubrinol acetate (**7**), and an unknown compound (**UC1**: T_R_ 14.0 min, UV λ_max_: 226, 267, 315, 366, 565 nm, M._f._ C_46_H_48_O_16_, M._wt._ 856) as major components together with orsellinic acid (**6**), hypomiltin (**14**), daldiniapyrone (**17**) (Hellwig et al. 2005; Stadler et al. 2007) as minor components, whereas four unknown compounds (**UC2**: T_R_ 13.20 min, UV λ_max_: 226, 267, 314, 362, 565 nm, M._f._ C_44_H_46_O_15_, M._wt._ 814; **UC3**: T_R_ 13.90 min, UV λ_max_: 243, 267, 316, 565 nm, M._f._ C_23_H_28_O_8_, M._wt._ 432; **UC4**: T_R_ 14.20 min, UV λ_max_: 227, 266, 316, 367, 565 nm, M._f._ C_46_H_48_O_16_, M._wt._ 856) ocurred as minor component (SI Figure S7).

**Material examined**: French Guiana, Sinnamary, Paracou, CIRAD field station, lowland rainforest, plot 3, on dead corticated branch, Jun. 2012, leg. G. Gruhn, GYJF 12180 (Holotype LIP).

***Hypoxylon ochraceotuberosum*** J. Fourn. & Lechat, Ascomycete.org 7(5): 184 (2015)

**Secondary metabolites**: Stromata revealed orsellinic acid (**6**), mitorubrinol acetate (**7**), rubiginosins A (**10**) (Hellwig et al. 2005; Stadler et al. 2007) and Z (**11**) (Becker et al. 2021) as major components together with an unknown compound (**UC1**: T_R_ 8.84 min, UV λ_max_: 222, 268, 352 nm, M._f._ C_23_H_22_O_9_, M._wt._ 442) as a minor component (SI Figure S8).

**Material examined**: French West Indies, Martinique, Case-Pilote, Fond Boucher, xero- to mesophilic forest, on dead corticated branch, Aug. 2013, leg. J. Fournier, MJF 13275 (Holotype LIP).

***Hypoxylon sepiaceum*** J. Fourn. & Lechat, Ascomycete.org 7(5): 202 (2015)

**Secondary metabolites**: Stromata contain orsellinic acid (**6**), mitorubrin (**2**), mitorubrinol acetate (**7**), rubiginosins A (**10**)/C (**18**), kasanocin C (**19**) (Hellwig et al. 2005; Stadler et al. 2007; Li et al. 2015), and unknown compounds (**UC1**: T_R_ 7.25 min, UV λ_max_: 202, 232, 283, 384 nm, M._f._ C_24_H_26_O_9_, M._wt._ 458; **UC2**: T_R_ 8.81 min, UV λ_max_: 221, 265, 302, 387 nm, M._f._ C_21_H_22_O_7_, M._wt._ 386) as major components as well as comazaphilone C (**20**) (Gao et al. 2011), cohaerin F (**21**) (Quang et al. 2006) as minor components (SI Figure S9).

**Material examined**: French West Indies, Martinique, Sainte-Marie, La Philippe forest, Trou Mulet, coastal mesophilic forest, on corticated log of Mahogany (*Swietenia macrophylla* King, Meliaceae), Aug. 2013, leg. J. Fournier, GYJF 13350 (Holotype LIP). French West Indies, Martinique, Fort-de-France, Absalon, trail to Plateau Michel, ca. 350 m, hygrophilic rainforest, on corticated branch, Aug. 2013, leg. J. Fournier, MJF 13225 (Paratype LIP). French West Indies, Martinique, ibid., corticated branch, Aug. 2013, leg. J. Fournier, MJF 13238 (Paratype LIP). French West Indies, Martinique, ibid., corticated branch, on effete stromata of *Camillea* sp., Jun. 2014, leg. J. Fournier, MJF 14042 (Paratype LIP). French West Indies, Martinique, Rivière-Pilote, Lépinay forest, mesophilic rainforest, on a corticated branch, Aug. 2013, leg. O. Roze, MJF 13108 (Paratype LIP).

***Hypoxylon paracouense*** J. Fourn. & Lechat, Ascomycete.org 7(2): 80 (2015)

**Secondary metabolites**: Stromata contain BNT (**1**) (Hellwig et al. 2005), azanigerone (**22**) (Zabala et al. 2012), and an unknown compound (**UC1**: T_R_ 10.20 min, UV λ_max_: 231, 325 nm, M._f._ C_23_H_30_O_9_, M._wt._ 450) as major components with another unknown compound (**UC2**: T_R_ 9.00 min, UV λ_max_: 226, 312, 438 nm, M._f._ C_25_H_28_O_11_, M._wt._ 505) as a minor component (SI Fig. S10).

**Material examined**: French Guiana, Sinnamary, Paracou, lowland rainforest, CIRAD field station, on dead corticated branch ca. 2 cm diam., Jun. 2012, leg. J. Fournier, GYJF 12185 (Holotype LIP).

***Hypoxylon rhombisporum*** J. Fourn. & Lechat, Ascomycete.org 7(2): 85 (2015)

**Secondary metabolites**: Stromata contain rickenyl E (**23**) and one unknown compound (**UC1**: T_R_ 8.40 min, UV λ_max_: 222, 298, 414, 566 nm, M._f._ C_20_H_12_O_4_, M._wt._ 316) as major components together with two unknown compounds (**UC2**: T_R_ 11.10 min, UV λ_max_: 225, 277, 441 nm, M._f._ C_21_H_34_O_3_, M._wt._ 334; **UC3**: T_R_ 14.90 min, UV λ_max_: 198, 225 nm, M._f._ C_21_H_36_O_2_, M._wt._ 320) as minor components (SI Figure S11).

**Material examined**: French Guiana, Régina, Nouragues natural reserve, Inselberg camp, trail to Pararé, ca. 1 km from the camp, rainforest, on bark of unidentified dead standing trunk, Jun. 2012, leg. J. Fournier, GYJF 12123 (Holotype LIP).

***Hypoxylon aureolimbatum*** J. Fourn. & Lechat, Ascomycete.org 7(2): 66 (2015)

**Secondary metabolites**: Stromata contain BNT (**1**), mitorubrinol (**3**), mitorubrinol acetate (**7**) (Hellwig et al. 2005; Stadler et al. 2007), and an unknown compound (**UC1**: T_R_ 7.20 min, UV λ_max_: 221, 291 nm; M._f._ C_21_H_16_O_9_, M._wt._ 412) as major components and an unknown compound (**UC2**: T_R_ 11.10 min, UV λ_max_: 226, 266, 439, 560 nm, M._f._ C_21_H_18_O_7_, M._wt._ 382) as a minor component.

**Material examined**: French Guiana, Sinnamary, Saint Elie track, lowland rainforest, on dead corticated branch, Apr. 2010, leg. C. Lechat, CLL 10008 (Holotype LIP).

***Hypoxylon cazenavei*** J. Fourn., Ascomycete.org, 6(3): 54 (2014)

**Secondary metabolites**: Stromata were shown to contain BNT (**1**) in addition to two unknown compounds (**UC1**: T_R_ 12.40 min, UV λ_max_: 226, 327 nm, M._f._ C_23_H_26_O_5_, M._wt._ 382; **UC2**: T_R_ 12.80 min, UV λ_max_: 226, 320 nm, M._f._ C_23_H_30_O_5_, M._wt._ 386) as major components. In addition, two unknown compounds (**UC3**: T_R_ 9.10 min, UV λ_max_: 226, 317, 439, 562 nm, M._f._ C_31_H_23_O_8_, M._wt._ 522; **UC4**: T_R_ 10.40 min, UV λ_max_: 224, 324 nm, M._f._ C_27_H_40_O_6_, M._wt._ 460) were present as minor components (SI Figure S12).

**Material examined**: France, Ariège, Rimont, Grand Bois, Combe Fourcade, 42°57’45.64”‘N, 1°17’38.04’‘ E, mixed beech forest, 700 m alt., Sept. 2011, on bark of a rotting trunk of *Fagus sylvatica*, associated with old, weathered stromata of *Jackrogersella cohaerens*, leg. J. Fournier, JF 11128 (Holotype LIP). France, Ariège, Rimont, Grand Bois, Combe Fourcade, 42°57’45.64’‘N, 1°17’38.04” E, mixed beech forest, 700 m alt., Oct. 2012, on bark of a rotting trunk of *Fagus sylvatica*, leg. J. Fournier, JF 12111 (Paratype LIP).

***Hypoxylon flavocremeum*** J. Fourn., M. Pélissier & Lechat, Ascomycete.org 6(4): 65 (2014)

**Secondary metabolites**: Stromata contain mitorubrinol acetate (**7**) (Hellwig et al. 2005; Stadler et al. 2007) together with two unknown compounds (**UC1**: T_R_ 8.14 min, UV λ_max_: 202, 234, 266, 301, 388 nm, M._f._ C_27_H_40_O_9_, M._wt._ 508; **UC2**: T_R_ 10.43 min, UV λ_max_: 218, 266, 348 nm, M._f._ C_23_H_24_O_9_, M._wt._ 444) while two other unknown compounds (**UC3**: T_R_ 10.43 min, UV λ_max_: 219, 296 nm, M._f._ C_29_H_40_O_9_, M._wt._ 532; **UC3**: T_R_ 10.67 min, UV λ_max_: 219, 296 nm, M._f._ C_29_H_42_O_9_, M._wt._ 534) as minor components (SI Figure S13).

**Material examined**: Mayotte, Trevani, trail from Mayco to Trevani, below Kangani pass, S 12° 44’ 34.0’’ E 45° 10’ 59.5’’, 100 m alt., on corticated branch of *Mangifera indica*, Mar. 2014, Maurice Pélissier, CLL 14017 (Holotype LIP, ex-type culture CBS 138643). Mayotte, Dzoumogné, S 12° 42’ 56.8’’ E 45° 06’ 24.0”, on corticated branch, Feb. 2014, Maurice Pélissier, MP 2014-081 (Paratype LIP). Mayotte, Trevani, Vanilla trail from Coconi to Chiconi, S 12° 50’ 13.7’’ E 45° 07’ 15.9’’, on corticated branch of presumably *Mangifera indica*, Apr. 2014, Maurice Pélissier, MP 2014-142 (Paratype LIP).

## 4. Discussion

The present study provides valuable additional information on the taxonomy of *Hypoxylon* and allies from the Caribbean that can be added to the excellent morphological descriptions and the designation of *H. perforatum* from USA as epitype (ex-type culture STMA 23134). The selected strain fits perfect due to its morphological characters and it clusters with high support (1/100) with another strain of *H. perforatum* CBS 115281, in the clade H7, where the *H. rubiginosum* complex is located. Interestingly, all species located in clade H7 that have been previously examined for secondary metabolites contain mitorubrin type azaphilones. Only the Chinese species included have not been studied by HPLC, but they have orange or yellow stromatal pigments.

The newly generated data are largely in agreement with the conclusions drawn previously from morphological traits, but chemotaxonomic data sometimes provided a higher resolution.

For example, *Hypoxylon dussii* morphologically resembles *H. investiens* (Fournier et al. 2015), and both taxa also cluster together in the phylogenetic tree with high support (1/100), establishing both as sister species of one another. Additionally, the HPLC profile of *H. dussii* showed the presence of BNT (**1**), mitorubrin (**2**), mitorubrinol (**3**), and an unknown major metabolite. However, previously studied specimens of *H. investiens* revealed the presence of BNT and daldinone A (Sir et al. 2016). Therefore, *Hypoxylon dussii* significantly differs from *H. investiens* in its chemotaxonomic traits.

On the other hand, the position of *H. sofaiense* was not resolved and clustered with *H. musceum* receiving low support (0.99/-) in a sister clade with *H. isabellinum, H. perforatum*, and *H. sporistriatatunicum* with high support (1/95). Despite the morphological resemblance between *H. sofaiense* and *H. perforatum* (Fournier et al. 2015), they revealed different metabolic patterns. Both species were shown to contain orsellinic acid (**6**) as a common major metabolite, but stroma extracts derived of *H. sofaiense* revealed mitorubrinol acetate (**7**), whereas *H. perforatum* was reported to contain hypomiltin (Hellwig et al. 2005). Hypomiltin (**14**) was also found in *H. sporistriatatunicum* (Cedeño-Sanchez et al. 2020), but not in *H. isabellinum* and *H. musceum* (Kuhnert et al. 2014). Mitorubrinol (**3**) was only found in *H. musceum* (Kuhnert et al. 2014). Although these azaphilones are rather similar in their chemical structures, the above examples demonstrate the utility of HPLC profiling to delineate distinct taxa in species complexes in *Hypoxylon*.

Among the specimens studied, two species were formerly provisionally placed in *Hypoxylon* due to strongly deviating morphological characters. Even though it has been clarified that teleomorph morphology alone is of subordinate importance, due to representing a highly variable feature in the stromatic Xylariales, and the modern family concept being largely defined by molecular and anamorphic data, we nevertheless want to point out these examples.

*Hypoxylon cypraeisporum* described by Fournier et al. (2015) differs from the other species of *Hypoxylon* by featuring ascospores with pitted epispores. This character is otherwise only present in *H. rubellum*, which *H. cypraeisporum* differs from by a distinct stromatal morphology and smaller ascospores (15.9–17.6 × 7.6–8.5 µm vs 16.5–21 × 7–8 µm) with perispore dehiscent in 10% KOH. Nevertheless, the discoid configuration of the ostiolar region, combined with the carbonaceous texture of the stromatal wall, and the presence of hinnulin A (**9**) as a major metabolite in the stromata of *H*. *cypraeisporum* suggests strong affinities with *Annulohypoxylon* or *Jackrogersella.*

In the case of *H. nudum*, no secondary metabolites or KOH-extractable stromatal pigments were detected in this study. This phenomenon of a lack of KOH-extractable pigments is not unique within the Hypoxylaceae; for instance, species of *Hypomontagnella* also exhibit this trait, with pigments only present in young stromata (Lambert et al. 2019).

Other noteworthy results observed in this study comprise the stromatal secondary metabolites of *H. verruciperisporium*, mitorubrinol acetate (**7**), rubiginosins A (**10**)/Z (**11**), rutilins A (**12**)/B (**13**) as major components. This species, compared to *H. erythostroma*, shares orsellinic acid (**6**), mitorubrinol (**3**), and mitorubrinol acetate (**7**) (Sir et al. 2016).

*Hypoxylon subtrugodes* is morphologically close to *H. trugodes* (Fournier et al. 2015), but both species drastically differ in their stromatal HPLC profiles. While *H. subtrugodes* contains BNT (**1**), daldinals A (**15**), and B (**16**) as major metabolites, *H. trugodes* was reported to contain orsellinic acid (**6**) and hypomiltin (**14**) in its stromata instead (Hellwig et al. 2005). These chemotaxonomic data are rather significant because the polyketide core structures of daldinials A and B have been mainly reported to occur in *Daldinia childae* and the *H. fuscum* complex (Stadler et al. 2014; Lambert et al. 2021). Instead, hypomiltin (a mitorubrin type azaphilone!) was detected exclusively in stromata derived from *Hypoxylon* spp. (described from *H. hypomiltum*), even though it is identical to mitorubrinol acetate except for the lack of a single double bond.

In the case of *H*. *arawakianum*, all detected metabolites were unknown, indicating the need for additional recollection, isolation, and structure elucidation of the associated compounds. It was not even possible to assign any similarity to other known metabolites of the Xylariales based on the data we got on the molecular sum formulae. Furthermore, this study underscores the critical role of molecular analysis in accurately identifying and elucidating the relationships among species of *Hypoxylon* and related genera. Overall, the present study lays an important fundament and contributes towards a comprehensive taxonomy of *Hypoxylon* and related secondary metabolism of this diverse genus.

Recently, another study on *Hypoxylon* from French Guiana was also published, which has also revealed a substantial diversity of the genus in a relatively small geographic area, including 13 new well-described species (Fournier et al. 2024). The authors have provided ITS data, so at least a primary barcode is available for many of the new taxa. However, the cultures they made apparently did not survive and are thus not available for multi-gene genealogies, phylogenomic studies and examination of their secondary metabolites. It should also be interesting to study these materials, and at least add chemotaxonomic traits. In general, these results taken together indicate that the diversity of *Hypoxylon* and other genera of the Hypoxylaceae in the Neotropics is higher than ever envisaged before. It should be worthwhile to conduct intensive fieldwork there and in other tropical areas, but the availability of cultures will be important for future studies.

## Supplementary Material

mafft_alig_2024.jpg
